# Nurses’ perception of patient safety culture and its relationship with adverse events: a national questionnaire survey in Iran

**DOI:** 10.1186/s12912-021-00571-w

**Published:** 2021-04-12

**Authors:** Edris Kakemam, Hojatolah Gharaee, Mohamad Reza Rajabi, Milad Nadernejad, Zahra Khakdel, Pouran Raeissi, Rohollah Kalhor

**Affiliations:** 1 Department of Health Services Management, School of Management and Medical Informatics, Tabriz University of Medical Sciences, Tabriz, Iran; 2 District Health Center of Hamadan City, Hamadan University of Medical Sciences, Hamadan, Iran; 3 Department Cardiology, Faculty of Medicine, Shahed University, Tehran, Iran; 4 Health Management and Economics Research Center, Iran University of Medical Sciences, Tehran, Iran; 5 Health Management and Economics Research Center, Isfahan University of Medical Sciences, Isfahan, Iran; 6 Department of Health Services Management, School of Health Management and Information Sciences, Iran University of Medical Sciences, Tehran, Iran; 7 Social Determinants of Health Research Center, Research Institute for Prevention of Non-Communicable Diseases, Qazvin University of Medical Sciences, Qazvin, Iran; 8 Health Services Management Department, School of Public Health, Qazvin University of Medical Sciences, Shahid Bahonar Blv, Qazvin, Iran

**Keywords:** Adverse events, Patient safety, Patient safety culture, Nurses, Iran

## Abstract

**Background:**

Patient safety culture is an important factor in determining hospitals’ ability to address and reduce the occurrence of adverse events (AEs). However, few studies have reported on the impact of nurses’ perceptions of patient safety culture on the occurrence of AEs. Our study aimed to assess the association between nurses’ perception of patient safety culture and their perceived proportion of adverse events.

**Methods:**

A cross-sectional survey was carried out among 2295 nurses employed in thirty-two teaching hospitals in Iran. Nurses completed the Persian version of the hospital survey of patients’ safety culture between October 2018 and September 2019.

**Results:**

Positive Response Rates of overall patient safety culture was 34.1% and dimensions of patient safety culture varied from 20.9 to 43.8%. Also, nurses estimated that the occurrence of six adverse events varied from 51.2–63.0% in the past year. The higher nurses’ perceptions of “Staffing”, “Hospital handoffs and transitions”, “Frequency of event reporting”, “Non-punitive response to error”, “Supervisor expectation and actions promoting safety”, “Communication openness”, “Organizational learning continuous improvement”, “Teamwork within units”, and “Hospital management support patient safety” were significantly related to lower the perceived occurrence at least two out of six AEs (OR = 0.69 to 1.46).

**Conclusions:**

Our findings demonstrated that nurses’ perception regarding patient safety culture was low and the perceived occurrence of adverse events was high. The research has also shown that the higher level of nurses’ perception of patient safety culture was associated with lowered occurrence of AEs. Hence, managers could provide prerequisites to improve patient safety culture and reduce adverse events through different strategies, such as encouraging adverse events’ reporting and holding training courses for nurses. However, further research is needed to assess how interventions addressing patient safety culture might reduce the occurrence of adverse events.

**Supplementary Information:**

The online version contains supplementary material available at 10.1186/s12912-021-00571-w.

## Background

Adverse events (AEs) have become one of the most serious threats for patient safety and quality of care in hospitals. The World Health Organization defines them as errors that occur during nursing care, which cause measurable injury or damage to patient, not related to the underlying disease [1]. Such events may involve errors of medication or equipment, delays in taking therapeutic choices, misdiagnosis, infection, loss of device, and others [2] which could have a negative impact on patients’ safety and quality of care.

In the US, medical error has ranked as the third most common cause of death [3]. Moreover, they cause disability, injury and even death amongst patients globally [4], besides of raising the medical and hospitalization costs in developing and developed countries [2, 5].

Even though there are many medical advances in treatment and diagnosis, AEs are still a huge problem for staff and patients because these treatments and diagnosis are often highly complex and can be affected by many different issues involving human error and hospital systems [2].

There is a growing body of literature that has estimated the AEs incidence rate in different countries. For instance, a recent systematic review has shown that 2.9 to 21.9% of patients have experienced AEs [2]. Kang, et al. (2016) estimated that 36–57% of nurses have reported at least one of four AEs in the last 12 months [6]. In another study in China, 47.8–75.6% of nurses reported that AEs had happened in the past year [7]. In a recent national study in Iran, 29.1% of nurses experienced AEs in the past six months [8]. According to a systematic review in 2019, the prevalence of AEs in Iran was 10 to 80% [5]. Another systematic review of 48 studies using the Global Trigger Tool revealed the occurrence of AEs varied between 7 and 40% [9].

We understand that errors can be unavoidable; they result from a broken barrier of the system that involve them since the buying of the acquisition of the material to its administration. Then, it does not work to blame individuals or a punitive conduct to avoid AEs. To improve patient safety is important to make emphasis to system improvement [10].

Experts in the field viewed patient safety as being at the center of healthcare quality. Hence, they carried out a huge amount of work to improve systems for patient safety, including the perception of the culture of patient safety by professionals. Patient safety culture defines “management and staff values, beliefs, and norms about what is important in a health care organization, how organization members are expected to behave, what attitudes and actions are appropriate or inappropriate, and what processes and procedures are rewarded and punished, concerning patient safety” [11].

In healthcare organizations, in particular, in the hospitals, the culture of patient safety relies on communications based on reciprocal trust, suitable information flows, organizational learning, common perceptions of the importance of safety, commitments of leadership, as well as management of the organization, and the presence of a non-punitive strategy to deal with the occurrence of AEs and error reporting [12].

Patient safety is a new concept in Iranian hospitals. Nevertheless, from 2009 in the Iranian healthcare system, initiatives such as hospital accreditation, clinical governance and patient safety friendly hospitals have been introduced as frameworks to improve safety and quality in-hospital care [13]. In Iranian hospitals, the lack of an active, systematic and national error reporting mechanism is a major problem causing not reporting errors being very common. As yet heath care workers report errors passively and voluntary through the manually reporting forms. In addition, to reduce adverse events the strategy of root cause analysis is implementing by hospitals of Iran [14].. Errors reporting by nurses not only included no incentive, but for some severe errors the offender may be blamed or punished by managers in many Iranian hospitals, without considering the reasons for such errors [15, 16]. Although hospitals provide patient safety training courses, there is no coherent curriculum in the field of patient safety in nursing education in universities [17].

Establishing patient safety culture at the heart of all healthcare settings has been suggested as the key factor in improving patient safety with the potential to stop errors from happening [18]. Some studies have conducted in the field of linking of nurses’ perception of patient safety culture with the occurrence of AEs. Wang et al. (2014) demonstrated the association of improvements in patient safety culture with a lower incidence of the AEs [7]. Hwang (2011) also reported the greater patient safety culture and fewer AEs [19]. Farup (2015) found that there was a negative correlation between AEs and patient safety culture [20]. Previous studies have found that developing a positive patient safety culture, such as a non-punitive environment, lead to voluntary reports of near misses and errors [21–23].

Although, several studies have reported the relationship between nurses’ perception of patient safety culture and the occurrence of AEs, up to now, there has been no study conducted in Iran. Most studies have also estimated the frequency of AEs at the level of the single units and based on reporting systems. Identifying the types of AEs and its prevalence has an important role in planning for prevention [5]. Unfortunately, despite the considerable damages caused by AEs, there is little evidence about the role and impact of patient safety culture in developing countries, and hence, these countries do not have a good understanding of the patient safety culture status in their hospitals [24].

Therefore, considering the importance of AEs and its relationship with patient safety culture, this study aims to assess the association between nurses’ perception of patient safety culture and the perceived proportion of AEs in teaching hospitals in Iran. The research questions were:

- What is the level of perception of patient safety culture among Iranian nurses working in teaching hospitals?
- What is the nurses’ perceived proportion of AEs?
- Does nurses’ perception of patient safety culture impact on their perception of the AEs?

Our research is based on the hypothesis that higher level of nurses’ perception of patient safety culture would be associated with lowered perceived of AEs.

## Methods

### Study design

A nationwide cross-sectional study was conducted between October 2018 and September 2019. 

### Setting

Iran includes 31 provincial centers (or capital), of which five centers randomly were selected. There are in total of 570 public hospitals throughout Iran. Of the 570 public hospitals, 150 are teaching hospitals. Only teaching hospitals were included in this study. The selected cities and hospitals were similar in terms of demographic and socioeconomic status. Convenient sampling was used to select teaching hospitals including 32 hospitals in selected centers. Tabriz (8 hospitals), Tehran (16 hospitals), Qazvin (6 hospitals), Esfahan (7 hospitals), Hamedan (7 hospitals) were included in this study. Out of 32 studied hospitals, seven hospitals were large hospitals (> 300 beds), 15 medium hospitals (100–300 beds) and ten small hospitals (less than 100 beds). The units included were general wards, intensive care units and emergency departments.

### Participants

The target population included nurses working in the units. The inclusion criteria to select the nurses were as follows: 1) The full-time nurses, 2) Nurses with more than one year’s work experience in the current hospital, 3) Working in the clinical post, and 4) Nurses who accepted to participate of the study. A convenience sampling method was applied to select nurses. Totally, 4500 questionnaires were distributed, 3450 (64.7%) nurses returned the questionnaires and 2295 (51.1%) questionnaires were completed accurately. The demographic information of both the complete and incomplete questionnaires was analyzed using χ^2^ tests, with no significant differences found.

### Instruments

Data were collected using the three following instruments:

#### Demographic questionnaire

Demographics and work variables of participants included questions related to gender, age, marital status, working unit, educational level, work experience (years), and work time (hours per week) and hospital size (number of beds).

#### Patient Safety culture 

We used the Persian version of Hospital Survey of Patients’ Safety Culture (HSOPSC), which was translated and modified to suit the Iranian system by Moghri et al. [25]. The HSOPSC (the original U.S. English version 2010) was developed and tested by the Agency for Healthcare Research and Quality in 2004 [11].

The HSOPSC consists of 42 items that measure 12 patient safety culture dimensions: “Communication openness” (3 items), “Feedback and communication about errors” (3 items), “Frequency of events reported” (3 items), “Handoffs and transitions” (4 items), “Management support for patient safety” (3 items), “Non-punitive response to error” (3 items), “Organizational learning/continuous improvement” (3 items), “Overall perception of patient safety” (4 items), “Staffing” (4 items), “Supervisor/manager expectations and actions promoting safety” (4 items), and “Teamwork across and within units” (4 items). All items measured were based on 5-point Likert response scales of agreement (strongly disagree to strongly agree) or frequency (never to always), so the mean score of each dimension could be calculated.

We calculated the positive response rate (PRR) to analyze the positive attitudes towards patient safety culture among nurses. First, a PRR for each item from the responses related to “strongly agree/agree” or “always/most of the time”. Therefore, for the calculation of the PRR of each of the dimensions, the initial phase has been the computation of the PRR for each item and subsequently calculation of the mean PRR among each item. Accordingly, it is possible to calculate the mean PRR of the overall patient safety culture [7]. PRRs of 75% and above, between 50 and 75% and less than 50% are considered as representing areas of strength, neutral and areas requiring improvement, respectively [26, 27]. The internal consistency reliability estimated with Cronbach’s coefficient alpha of the original English version ranged between 0.63 and 0.84 [11], whereas for the Persian version, it ranged between 0.57 and 0.80 [25]. In this study, Cronbach’s coefficient alpha values ranged from 0.76 to 0.82.

#### Adverse events

To collect AEs data, a range of methods - all of which have their own advantages and disadvantages - are routinely used: the review of the nursing or medical record, reporting systems, direct observations, patient interviews, and estimate of the nurses [7, 28]. For this study, we looked at the estimates of AEs at a nurse’s individual level. This method meant we could gather lots of accurate data over a short period of time without fear of punishment [29]. The disadvantages of using this method are well documented and include respondent bias and recall bias [28]. However, it has been found that the nurses’ estimated “patient fall” frequency over one year was more accurate than systematically assessed data and was concordant with continuously assessed data over the same time frame [30]. The usefulness and accuracy of this method have been confirmed in other researches [].

We chose to focus on the six AEs which happen most frequently in hospitals and nurses are required to report on [7, 14]: Pressure ulcer, Patient fall, Adverse drug event, Surgical wound infection, Patients or their family complaints, and Infusion or transfusion reaction. We asked the nurses to indicate whether they had experienced AEs in the last year. We used a 7-level rating system estimated by nurses. The AEs frequency rated “everyday = 6”, “several times a week = 5”, “once a week = 4”, “several times a month = 3”, “once a month or less = 2”, “several times a year = 1”, and “never happen = 0” in the past year using a 7-level rating scale estimated by nurses.

### Data collection

The data was collected by all authors and three trained research assistants during morning and evening shifts. Before completing the questionnaire by the nurses, the investigators informed them about the purpose and significance of the study. Our data collection method was to ask nurses to fill out a paper-based survey, either at home or at work. Beforehand we worked closely with the hospital administration to plan and co-ordinate data collection and ensure we achieved a maximum response rate. We didn’t provide any incentives for nurses to fill out the questionnaire.

### Data analysis

The participants’ demographic characteristics, nurses’ perception of patient safety culture dimensions, and the frequency of AEs were described using descriptive statistic indicators such as frequency, percentage, standard deviation (SD), and mean. Sex categories of frequencies of AEs were entered as response variables after it was dichotomized into “never happened = 0” (response of never happened) and “had happened = 1” (response of “several times a year”, “once a month or less”, “several times a month”, “once a week”, “several times a week” or “everyday”) based on a previous study [7].

In the next stage, the logistic regression analysis models were used for the determination of the association of explanatory variables (twelve dimensions of the patient safety culture) with the variable of response (AEs). For the first step, bivariate regression models were performed for each AE as the dependent variable and 12 patient safety culture dimensions as independent variables. For the second step, we used multiple logistic regression models with one type of AE and 12 patient safety culture dimensions alongside with the control of all nurses’ demographic variables. Significance level was considered 0.05.

## Results

We presented sample characteristics in Table 1. Most of the participants were female (79.7%) and married (51.8%), and had a bachelor of science in nursing degree (77.4%). Nurses were between 21 and 63 years old, and the mean age was 34.14 (SD = 7.07) years. The largest age group was 31–40 years old (46.6%). More than half of the nurses worked in general wards (56.8%). Most of the nurses (65.3) worked 44 or fewer hours per week. Nearly 70% of nurses had less than 11 years’ experience with an average experience of 8.96 (SD = 6.77) years.

**Table 1:** Demographic characteristics of nurses (*n* = 2295)

Variables	N (%)
Gender	
*Male*	468 (20.4)
*Female*	1827 (79.6)
Marital status	
*Single*	1106 (48.2)
*Married*	1189 (51.8)
Age (in years)	
*21–30*	840 (36.6)
*31–40*	1070 (46.6)
*> 40*	385 (16.8)
Experience (in years)	
*1–5*	922 (40.2)
*6–10*	615 (26.8)
*> 10*	758 (33.0)
Hours worked per week	
*≤ 44*	1498 (65.3)
*> 44*	797 (34.7)
Education in nursing	
*Bachelor degree*	1777 (77.4)
*Master degree or PhD*	518 (22.6)
Current work unit	
*Critical care units*^a^	533 (23.2)
*Emergency department*	458 (20.0)
*General wards*^b^	1304 (56.8)
Number of beds	
*≤ 200*	856 (37.3)
*200–499*	1040 (45.3)
*> 499*	399 (17.4)

^a^-CCU, ICU, NICU, PICU, Post ICU; ^b^-Internal, surgical, obstetrics, pediatrics, and Orthopaedics, cardiology, psychiatry

The PRRs and mean scores of patient safety culture dimensions and the overall score are presented in Table 2. Mean (SD) scores for patient safety culture dimensions ranged from 3.31 (0.73) to 2.63 (0.82) and the PRRs ranged from 20.9 to 43.8%. The PRRs of patient safety culture dimensions were all less than 50% and the overall PRR was 34.1%. The PRR of “Teamwork within units” (PRR = 43.8%) was the highest followed by “Organizational Learning/ Continuous Improvement” (PRR = 42.7%). The PRR of “Hospital Handoffs and Transitions” (PRR = 20.9%) was the lowest.

**Table 2:** The mean scores and positive response rate of patient safety culture

Patient safety culture dimensions	Mean (SD)	PRR^**a**^%	Judgment
Organizational learning continuous improvement	3.31 (0.73)	42.7	Requiring improvement
Teamwork within units	3.27 (0.78)	43.8	Requiring improvement
Feedback and communication about error	3.24 (0.74)	41.1	Requiring improvement
Non-punitive response error	3.14 (0.82)	38.4	Requiring improvement
Frequency event reporting	3.10 (0.74)	37.7	Requiring improvement
Hospital management supports	3.09 (0.63)	34.9	Requiring improvement
Staffing	3.02 (0.66)	34.7	Requiring improvement
Teamwork across hospital units	2.97 (0.54)	29.7	Requiring improvement
Overall perceptions of safety	2.93 (0.54)	31.5	Requiring improvement
Communication openness	2.84 (0.64)	27.2	Requiring improvement
Supervisor/manager expectations action promoting safety	2.71 (0.65)	26.5	Requiring improvement
Hospital handoffs and transitions	2.63 (0.82)	20.9	Requiring improvement
Overall patient safety culture	3.02 (0.34)	34.1	Requiring improvement

^a^ Positive Response Rate (PRR)

Table 3 reports the prevalence of AEs. The majority of participants reported that six AEs happened “several times a year”, followed by “once a month or less”. A few participants stated that AEs happened “once a week”, “several times a week” and “every day”. Only 3.9% of nurses reported that Adverse drug events occurred “several times a week” and 7.8% nurses reported it occurred “every day”.

**Table 3:** Estimated adverse events in the past year among nurses (n = 2295)

Adverse events	Never happened N(%)	Several times a year N(%)	Once a month or less N(%)	Several times a month N(%)	Once a week N(%)	Several times a week N(%)	Everyday N(%)
Pressure ulcer	1065 (46.4)	675 (29.4)	273 (11.9)	122 (5.3)	46 (2.0)	81 (3.5)	33 (1.3)
Patient fall	1125 (49.0)	792 (34.5)	215 (9.4)	47 (2.0)	31 (1.4)	54 (2.4)	31 (1.4)
Adverse drug events	900 (39.2)	819 (35.7)	338 (14.7)	100 (4.4)	37 (1.6)	58 (2.5)	43 (1.9)
Surgical wound infection	1037 (45.2)	681 (29.7)	286 (12.5)	149 (6.5)	51 (2.2)	42 (1.8)	49 (2.1)
Infusion or transfusion reaction	1121 (48.8)	737 (32.1)	241 (10.5)	74 (3.2)	29 (1.3)	43 (1.9)	50 (2.2)
Patients or family complaints	849 (37.0)	747 (32.5)	299 (13.0)	157 (6.8)	74 (3.2)	103 (4.5)	66 (2.9)

After merging the six kinds of AEs frequency into a binomial variable, the nurses reported AEs occurrence of 63.0% (Patients or their family complaints) to 51.2% (Patient fall) during the past year; 60.8% Adverse drug events, 54.8% Surgical wound infection, 53.6% Pressure ulcer, and 51.2% Infusion or transfusion reaction.

The results of multiple logistic regression models are presented in Table 4. Results for the unadjusted model and an adjusted model for potentially confounding demographic factors are reported. After controlling the confounding effects of demographic factors, the results did not change significantly. The full results of logistic regression models are shown in the additional file 1. “Frequency of event reporting” and “Non-punitive response to error” associated with all the AEs. “Teamwork across Hospital Units”, “Feedback Communication about Error”, and “Overall Perception of Safety culture” were not correlated with any of the AEs. The variance of OR is from 0.69 (the odds of patients or their family complaints were 69.0% as large for each unit increase in the score of “Hospital Handoffs and Transitions”) to 1.46 (the odds of adverse drug events were 146% as large for each unit increase in the score of “Non-punitive Response to Error”).

**Table 4:** Bivariate and multiple logistic regression results of the relationship between dimensions of patient safety culture and adverse events

	Unadjusted (bivariate) models		Adjusted (multiple) models	
	OR (95% CI)	***p***	OR (95% CI)	***p***
***Pressure ulcer***				
Organizational learning-continuous improvement	0.67 [0.58–0.78]	*< 0.001*	0.69 [0.59–0.81]	*< 0.001*
Non-punitive response to error	1.34 [1.19–1.50]	*< 0.001*	1.27 [1.12–1.43]	*< 0.001*
Staffing	0.82 [0.71–0.95]	*0.009*	0.79 [0.68–0.92]	*0.003*
Hospital handoffs and transitions	0.70 [0.62–0.80]	*< 0.001*	0.75 [0.66–0.85]	*< 0.001*
Frequency of event reporting	0.76 [0.67–0.86]	*< 0.001*	0.77 [0.68–0.88]	*< 0.001*
***Patient fall***				
Organizational learning-continuous improvement	0.72 [0.62–0.83]	*< 0.001*	0.75 [0.64–0.87]	*< 0.001*
Teamwork within units	1.18 [1.03–1.36]	*0.021*	1.16 [1.01–1.35]	*0.041*
Non-punitive response to error	1.11 [0.99–1.24]	*0.080*	1.14 [1.01–1.28]	*0.036*
Staffing	0.76 [0.65–0.88]	*< 0.001*	0.74 [0.64–0.87]	*< 0.001*
Hospital management support for patient safety	0.79 [0.68–1.93]	*0.003*	0.75 [0.64–0.88]	*0.001*
Frequency of event reporting	0.85 [0.75–0.95]	*0.006*	0.88 [0.78–0.99]	*0.044*
***Adverse drug events***				
Supervisor expectation & actions promoting safety	0.77 [0.66–0.90]	*0.001*	0.79 [0.68–0.93]	*0.005*
Teamwork within units	1.28 [1.11–1.49]	*0.001*	1.29 [1.11–1.50]	*0.001*
Communication openness	0.80 [0.68–0.94]	*0.007*	0.78 [0.68–0.94]	*0.007*
Non-punitive response to error	1.49 [1.32–1.68]	*< 0.001*	1.46 [1.29–1.65]	*< 0.001*
Staffing	0.79 [0.68–0.93]	*0.003*	0.77 [0.65–0.89]	*0.001*
Hospital handoffs and transitions	0.76 [0.66–0.86]	*< 0.001*	0.79 [0.69–0.90]	*< 0.001*
Frequency of event reporting	0.76 [0.67–0.86]	*< 0.001*	0.78 [0.68–0.88]	*< 0.001*
***Surgical wound infection***				
Communication openness	0.80 [0.69–0.94]	*0.006*	0.79 [0.68–0.93]	*0.004*
Non-punitive response to error	1.36 [1.21–1.53]	*< 0.001*	1.35 [1.20–1.52]	*< 0.001*
Staffing	0.81 [0.70–0.94]	*0.004*	0.78 [0.67–0.91]	*0.002*
Hospital management support for patient safety	0.81 [0.69–0.95]	*0.009*	0.80 [0.68–0.94]	*0.007*
Hospital handoffs and transitions	0.84 [0.74–0.95]	*0.005*	0.86 [0.76–0.98]	*0.025*
Frequency of event reporting	0.78 [0.69–0.88]	*< 0.001*	0.77 [0.68–0.88]	*< 0.001*
***Infusion or transfusion reaction***				
Supervisor expectation & actions promoting safety	0.82 [0.71–0.96]	*0.011*	0.84 [0.72–0.97]	*0.022*
Non-punitive response to error	1.25 [1.12–1.40]	*< 0.001*	1.23 [1.09–1.38]	*0.001*
Hospital handoffs and transitions	0.80 [0.71–0.90]	*< 0.001*	0.82 [0.73–0.94]	*0.003*
Frequency of event reporting	0.74 [0.65–0.83]	*< 0.001*	0.75 [0.66–0.85]	*< 0.001*
***Patients or their family complaints***				
Supervisor expectation & actions promoting safety	0.69 [0.59–0.81]	*< 0.001*	0.73 [0.62–0.85]	*< 0.001*
Communication openness	0.84 [0.71–0.98]	*0.032*	0.84 [0.71–0.99]	*0.034*
Non-punitive response to error	1.39 [1.23–1.57]	*< 0.001*	1.35 [1.12–1.53]	*< 0.001*
Staffing	0.87 [0.75–1.02]	*0.086*	0.83 [0.71–0.98]	*0.026*
Hospital handoffs and transitions	0.68 [0.59–0.77]	*< 0.001*	0.69 [0.60–0.79]	*< 0.001*
Frequency of event reporting	0.82 [0.72–0.93]	*0.002*	0.85 [0.74–0.97]	*0.015*

*CI* Confidence interval, *OR* Odds ratios

## Discussion

The present study is the first comprehensive research to determine the relationship between nurses’ perception of patient safety culture and the perceived occurrence of AEs among Iranian nurses at an individual level.

Based on the results, the overall score of PRR for patient safety culture was 34.1%. Moreover, the PRR scores for all dimensions of patient safety culture were lower than 50%. These findings show that patient safety culture in teaching hospitals is poor and needs urgent improvement. Decision-makers must focus on the areas such as teamwork across hospital units, overall perceptions of safety and communication openness. This is possibly because of patient safety culture is a relatively new concept in Iranian hospitals and has not been fully recognized. In this regard, a study in Iran has reported several challenges and obstacles to implement and integrate a positive safety culture. Those challenges included inadequate organizational infrastructure, insufficient leadership effectiveness, inadequate efforts to keep pace with national and international standards, and overshadowed values of team participation [17].

Recently, a systematic review revealed that the patient safety culture level in Iranian hospitals is low which is in line with our findings [24]. These findings contradicted with Raeissi et al [32] and Khoshakhlagh et al [33] studies’ results, which revealed that PRR scores of patient safety culture dimensions in the investigated hospitals is higher than the findings in our study. In addition, the overall level of the patient safety culture of this study is lower when compared with studies in other countries; 46.7% in Ethiopia [26], 54.7% in China [7], 52.9% in Taiwan [34], 52.2% in the Netherlands [35], 52.9% in Jordan [36], 51.8% in Japan [34], 54% in Palestine [37], 62% in the USA [35], and 49.1% in Saudi Arabia [27]. The differences are possibly caused by variations in organizational as well as cultural behaviours which affect patient safety perception in each country. It is possible that such countries had better organizational commitment, management value, leadership and also relationships between hospital personnel. Another probable reason could be the developed economy of these countries, leading to many countries tackling patient safety issues earlier in comparison with Iran. Regular training courses which are monitored and constantly improved would emphasise the importance of teamwork and thereby help nurses improve patient safety culture.

“Hospital Handoffs and Transitions” was the lowest-rated dimensions for patient safety culture. This finding is consistent with the results of studies carried out in Lebanon [38], Jordan [36], Japan [34], and Ethiopia [39]. Besides, a systematic review showed that in 36% of the reviewed studies (*N* = 12) the “Handoffs and transitions” dimension rated as weak [40].

Health organisations often target ‘hospital handoffs and transitions’ for quality improvement because hospitals experience safety incidents in this high-risk area, leading to important information being lost and patient care being fragmented [41].

One of the other dimensions that had low PRR was the “Supervisor/Manager Expectations Action Promoting Safety” (second lowest in the present research). This result was in accordance with other studies’ findings. One of these studies, performed in Iran, suggested that supervisor/manager expectations and actions promoting patient safety is necessary to improve patient safety culture in hospitals [33]. In order to increase and improve hospitals’ safety culture, we need to see a shift in staff’s values, beliefs, and behaviour which needs to match expected values of patient safety culture. However, for this to happen, senior executives, leaders, and supervisors need to support and help drive change [38].

Our study results demonstrated that the nurse-reported occurrence of AEs is high with 51.2–63.0% of nurses experienced the occurrence of such AEs over the last year. It is imperative to have accurate monitoring of in-hospital AEs, including via retrospective record reviews, in order to implement and evaluate evidence-based strategies to reduce AEs and ultimately, patient harm. Moreover, it is necessary that the nurses improve their communication skills and hospital managers establish an electronic health record mechanism for detecting and monitoring the AEs.

In addition, there are three other comparable studies, all of which found the same levels of AEs reported by nurses as our study did. Abadi et al. (2017) found between 59 and 76% of nurses have experienced at least one of six defined AEs [14]. In a recent study in Iran 48.0% of nurses had experienced adverse events in the past 6 months [42]. Kang, et al (2016) reported 36–57% rates of incidence in at least one of four AEs in the past year [6]. A study conducted in China has estimated the nurse-reported occurrence of AEs as between 47.8–75.6% in the past year [7]. Kakemam et al. found that 29.1% of Iranian nurses experienced AEs in the past six months [8]. A systematic review has reported the occurrence of the AEs between 2.9–21.9% [2]. The reasons for the high prevalence of AEs among nurses can due to workload, inappropriate shifts, longer work hours [43], high stress and long work hours that can make the occurrence of AEs [8].

Our study confirms the research hypothesis that higher level of nurses’ perception of patient safety culture was associated with lowered perceived of AEs. According to our findings in the multiple logistic models, the higher nurses’ perceptions of “Staffing”, “Hospital handoffs and transitions”, “Frequency of event reporting”, “Non-punitive response to error”, “Supervisor expectation and actions promoting safety”, “Communication openness”, “Organizational learning continuous improvement”, “Teamwork within units”, and “Hospital management support patient safety” were significantly related to lower the perceived occurrence at least two out of six AEs. Surprisingly, a study conducted in Norway demonstrated that there was an inverse association between patient safety culture and AEs [20]. In a study of Mardon et al. (2010) they observed that those hospitals which scored higher on the patient safety culture survey reported fewer cases of AEs [44]. Also, another study in China showed the relationship between improvements in the patient safety culture with a decreased incidence of AEs [7]. A study in Palestinian hospitals found that in units where the staff have more positive patient safety culture perception, less AEs were seen [37]. Another study examined the relationship between patient safety culture and missed nursing care, showed higher ratings of patient safety culture were associated with less missed nursing care [22]. Also, a study conducted in Iran, showed that higher level of patient safety culture was associated with higher intention to report errors [16].

### Strengths and limitations

This research, to our knowledge, was the first to explore the relationship of the perception of nurses of patient safety culture and the perceived proportion of AEs in the hospital context in Iran. Though the research empowered by numerous advantages such as multi-site settings and a big sample size with thirty-two of the teaching hospitals in Iran taking part, several shortcomings need to be considered when interpreting the findings. Firstly, we used convenience sampling methods for recruiting nurses. However, multiple centers were selected to increase the generalizability of present results and our sample represented the majority of the teaching hospitals in Iran. Secondly, the findings of the study relies on perceptions of both the patients safety culture and the occurrence of AEs. Nurses may over-or underestimate the numbers AEs or patient safety culture items because of fear of punishment by hospital management. Thirdly, the cross-sectional design of this study, only provided a ‘snapshot’, limiting conclusions of causality. Further research, using other designs such as longitudinal and controlled studies, is needed to determine the effectiveness of patient safety culture and further evaluate causality relationships. Finally, this study chose the nurses’ estimates to assess the frequencies of AEs which may not be accurate. Evans (2009) found that many AEs are not reported, for a range of reasons [45]. Nevertheless, Barbara et al. suggest that estimates of the frequency of AEs over the last 12 months are reliable. Collecting actual AEs data through the hospitals would increase the reliability of the data and provide more meaningful results from which to take action.

## Conclusions

This study examined the relationship between nurses’ perception of patient safety culture and the perceived proportion of AEs in teaching hospitals in Iran. Our findings illustrated that nurses’ perception of the patient safety culture was weak and the perceived proportion of AEs reported by nurses was high over the last year. Our study revealed that higher level of nurses’ perception of patient safety culture was associated with lowered occurrence of AEs. In particular, staffing, hospital handoffs and transitions, frequency of event reporting, non-punitive response to error, supervisor expectation and actions promoting safety, communication openness, organizational learning continuous improvement, teamwork within units and hospital management support patient safety are important factors of patient safety culture which associated with the occurrence of AEs. Therefore, the conventional culture of blaming or punishing healthcare professionals for AEs could be replaced with a non-punitive culture to develop nurse’s initiative to report AEs voluntarily. Hospitals need to develop interventions to improve patient safety culture. In addition, the nurses need to improve their communication skills and hospital managers need to establish an electronic health record mechanism for detecting and monitoring the AEs.

## Supplementary Information

**Additional file 1:** Table 4**Full.** Bivariate and multiple logistic regression results of the impact of patient safety culture on AEs

## Data Availability

The datasets analyzed during the current study are available from the corresponding author on reasonable request.

## Abbreviations

AEs: Adverse events
HSOPSC: Hospital Survey of Patients’ Safety Culture
PRR: Positive response rate
SD: Standard deviation
CI: Confidence interval
OR: Odds ratios

## References

[1] de Vries EN, Ramrattan MA, Smorenburg SM, Gouma DJ, Boermeester MA (2008). The incidence and nature of in-hospital adverse events: a systematic review. BMJ Qual Saf.

[2] Schwendimann R, Blatter C, Dhaini S, Simon M, Ausserhofer D (2018). The occurrence, types, consequences and preventability of in-hospital adverse events–a scoping review. BMC Health Serv Res.

[3] Makary MA, Daniel M (2016). Medical error—the third leading cause of death in the US. Bmj.

[4] Safety WP (2009). Global priorities for patient safety research.

[5] Vaziri S, Fakouri F, Mirzaei M, Afsharian M, Azizi M, Arab-Zozani M (2019). Prevalence of medical errors in Iran: a systematic review and meta-analysis. BMC Health Serv Res.

[6] Kang J-H, Kim C-W, Lee S-Y (2016). Nurse-perceived patient adverse events depend on nursing workload. Osong Public Health Res Perspect.

[7] Wang X, Liu K (2014). You L-m, Xiang J-g, Hu H-g, Zhang L-f, et al. the relationship between patient safety culture and adverse events: a questionnaire survey. Int J Nurs Stud.

[8] Kakemam E, Kalhor R, Khakdel Z, Khezri A, West S, Visentin D, Cleary M (2019). Occupational stress and cognitive failure of nurses and associations with on self-reported adverse events: a national cross-sectional survey. J Adv Nurs.

[9] Hibbert PD, Molloy CJ, Hooper TD, Wiles LK, Runciman WB, Lachman P, Muething SE, Braithwaite J (2016). The application of the global trigger tool: a systematic review. Int J Qual Health Care.

[10] Reason J (2000). Human error: models and management. BMJ: British Medical Journal.

[11] Sorra JS, Dyer N (2010). Multilevel psychometric properties of the AHRQ hospital survey on patient safety culture. BMC Health Serv Res.

[12] Sandars J, Cook G (2009). ABC of patient safety: John Wiley & Sons.

[13] Azami-Aghdash S, Azar FE, Rezapour A, Azami A, Rasi V, Klvany K (2015). Patient safety culture in hospitals of Iran: a systematic review and meta-analysis. Med J Islam Repub Iran.

[14] Abadi MBH, Akbari H, Akbari H, Gholami-Fesharaki M, Ghasemi M (2017). The association of nursing workloads, organizational, and individual factors with adverse patient outcome. Iran Red Crescent Med J.

[15] Hashemi F, Nasrabadi AN, Asghari F. Factors associated with reporting nursing errors in Iran: a qualitative study. BMC Nurs 2012;11(1):1–8, DOI: 10.1186/1472-6955-11-20. 10.1186/1472-6955-11-20 PMC3534596 23078517

[16] Chegini Z, Kakemam E, Jafarabadi MA, Janati A (2020). The impact of patient safety culture and the leader coaching behaviour of nurses on the intention to report errors: a cross-sectional survey. BMC Nurs.

[17] Farokhzadian J, Nayeri ND, Borhani F (2018). The long way ahead to achieve an effective patient safety culture: challenges perceived by nurses. BMC Health Serv Res.

[18] Reason J (1995). Understanding adverse events: human factors. BMJ Qual Saf.

[19] Hwang JI, Hwang EJ (2011). Individual and work environment characteristics associated with error occurrences in Korean public hospitals. J Clin Nurs.

[20] Farup PG (2015). Are measurements of patient safety culture and adverse events valid and reliable? Results from a cross sectional study. BMC Health Serv Res.

[21] Burlison JD, Quillivan RR, Kath LM, Zhou Y, Courtney SC, Cheng C, et al. A multilevel analysis of US hospital patient safety culture relationships with perceptions of voluntary event reporting. J Patient Saf. 2016;16(3):187–193. 10.1097/PTS.0000000000000336 PMC5415419 27820722

[22] Hessels AJ, Paliwal M, Weaver SH, Siddiqui D, Wurmser TA (2019). Impact of patient safety culture on missed nursing care and adverse patient events. J Nurs Care Qual.

[23] Richter JP, McAlearney AS, Pennell ML (2015). Evaluating the effect of safety culture on error reporting: a comparison of managerial and staff perspectives. Am J Med Qual.

[24] Behzadifar M, Behzadifar M, Jahanpanah F, Bragazzi NL (2019). Patient safety culture assessment in Iran using the “hospital survey on patient safety culture” tool: a systematic review and meta-analysis. Clin Epidemiol Glob Health.

[25] Moghri J, Arab M, Saari AA, Nateqi E, Forooshani AR, Ghiasvand H, Sohrabi R, Goudarzi R (2012). The psychometric properties of the Farsi version of “hospital survey on patient safety culture” in Iran’s hospitals. Iran J Public Health.

[26] Wami SD, Demssie AF, Wassie MM, Ahmed AN (2016). Patient safety culture and associated factors: a quantitative and qualitative study of healthcare workers’ view in Jimma zone hospitals, Southwest Ethiopia. BMC Health Serv Res.

[27] Alquwez N, Cruz JP, Almoghairi AM, Al-otaibi RS, Almutairi KO, Alicante JG (2018). Nurses’ perceptions of patient Safety culture in three hospitals in Saudi Arabia. J Nurs Scholarsh.

[28] Cina-Tschumi B, Schubert M, Kressig RW, De Geest S, Schwendimann R (2009). Frequencies of falls in Swiss hospitals: concordance between nurses’ estimates and fall incident reports. Int J Nurs Stud.

[29] Stratton KM, Blegen MA, Pepper G, Vaughn T (2004). Reporting of medication errors by pediatric nurses. J Pediatr Nurs.

[30] Manojlovich M, DeCicco B (2007). Healthy work environments, nurse-physician communication, and patients’ outcomes. Am J Crit Care.

[32] Raeissi P, Reisi N, Nasiripour AA (2018). Assessment of patient safety culture in Iranian academic hospitals: strengths and weaknesses. J Patient Saf.

[33] Khoshakhlagh AH, Khatooni E, Akbarzadeh I, Yazdanirad S, Sheidaei A (2019). Analysis of affecting factors on patient safety culture in public and private hospitals in Iran. BMC Health Serv Res.

[34] Fujita S, Seto K, Kitazawa T, Matsumoto K, Hasegawa T (2014). Characteristics of unit-level patient safety culture in hospitals in Japan: a cross-sectional study. BMC Health Serv Res.

[35] Wagner C, Smits M, Sorra J, Huang C (2013). Assessing patient safety culture in hospitals across countries. Int J Qual Health Care.

[36] Khater W, Akhu-Zaheya L, Al-Mahasneh S, Khater R (2015). Nurses' perceptions of patient safety culture in J ordanian hospitals. Int Nurs Rev.

[37] Najjar S, Nafouri N, Vanhaecht K, Euwema M (2015). The relationship between patient safety culture and adverse events: a study in Palestinian hospitals. Safety in Health.

[38] El-Jardali F, Jaafar M, Dimassi H, Jamal D, Hamdan R (2010). The current state of patient safety culture in Lebanese hospitals: a study at baseline. Int J Qual Health Care.

[39] Mekonnen AB, McLachlan AJ, Jo-anne EB, Mekonnen D, Abay Z (2017). Hospital survey on patient safety culture in Ethiopian public hospitals: a cross-sectional study. Safety Health..

[40] Reis CT, Paiva SG, Sousa P (2018). The patient safety culture: a systematic review by characteristics of hospital survey on patient safety culture dimensions. Int J Qual Health Care.

[41] Lee S-H, Phan PH, Dorman T, Weaver SJ, Pronovost PJ (2016). Handoffs, safety culture, and practices: evidence from the hospital survey on patient safety culture. BMC Health Serv Res.

[42] Kakemam E, Hajizadeh A, Azarmi M, Zahedi H, Gholizadeh M, Roh YS. Nurses' perception of teamwork and its relationship with the occurrence and reporting of adverse events: a questionnaire survey in teaching hospitals. J Nurs Manag. 2021. 10.1111/jonm.13257. 10.1111/jonm.13257 33480125

[43] Kakemam E, Raeissi P, Raoofi S, Soltani A, Sokhanvar M, Visentin DC, Cleary M (2019). Occupational stress and associated risk factors among nurses: a cross-sectional study. Contemp Nurse.

[44] Mardon RE, Khanna K, Sorra J, Dyer N, Famolaro T (2010). Exploring relationships between hospital patient safety culture and adverse events. J Patient Saf..

[45] Evans J (2009). Prevalence, risk factors, consequences and strategies for reducing medication errors in Australian hospitals: a literature review. Contemp Nurse.

