# Supplementary material for: Nurses’ perception of patient safety culture and its relationship with adverse events: a national questionnaire survey in Iran
Source: BMC Nurs. 2021 Apr 12;20:60. doi: 10.1186/s12912-021-00571-w (PMC8042945; doi:10.1186/s12912-021-00571-w)
Supplement: Supplementary file 1 — Additional file 1: Table 4Full. Bivariate and multiple logistic regression results of the impact of patient safety culture on AEs [file 12912_2021_571_MOESM1_ESM.docx]

**Supplementary Material**

**Table 4 Full. Bivariate and multiple logistic regression results of the impact of patient safety culture on AEs**

|  | Unadjusted (bivariate)) models | | Adjusted (multiple)) models | |
| --- | --- | --- | --- | --- |
|  | OR (95% CI) | *P* | OR (95% CI) | *P* |
| **Pressure Ulcer** | | | | |
| Supervisor Expectation & Actions Promoting Safety | 0.92 [0.79-1.07] | 0.281 | 0.99 [0.84-1.16] | 0.870 |
| Organizational Learning-Continuous Improvement | 0.67 [0.58-0.78] | *<0.001* | 0.69 [0.59-0.81] | *<0.001* |
| Teamwork Within Units | 1.15 [1.02-1.35] | 0.069 | 1.14 [0.99-1.32] | 0.079 |
| Communication Openness | 0.95 [0.82-1.12] | 0.551 | 0.97 [0.83-1.14] | 0.725 |
| Feedback and Communication About Error | 1.00 [0.86-1.15] | 0.954 | 0.96 [0.83-1.12] | 0.623 |
| Non-punitive Response to Error | 1.34 [1.19-1.50] | *<0.001* | 1.27 [1.12-1.43] | *<0.001* |
| Staffing | 0.82 [0.71-0.95] | 0.009 | 0.79 [0.68-0.92] | 0.003 |
| Hospital Management Support for Patient Safety | 0.93 [0.80-1.10] | 0.400 | 0.89 [0.75-1.04] | 0.142 |
| Teamwork Across Hospital Units | 0.93 [0.77-1.13] | 0.458 | 0.95 [0.78-1.15] | 0.570 |
| Hospital Handoffs and Transitions | 0.70 [0.62-0.80] | *<0.001* | 0.75 [0.66-0.85] | *<0.001* |
| Overall Perceptions of Safety | 1.09 [0.91-1.30] | 0.339 | 1.00 [0.84-1.20] | 0.963 |
| Frequency of Event Reporting | 0.76 [0.67-0.86] | *<0.001* | 0.77 [0.68-0.88] | *<0.001* |
| Gender (reference : *Male*) |  |  | 1.52 [1.21-1.89] | *<0.001* |
| Marital status (reference : *Single*) |  |  | 0.92 [0.75–1.11] | 0.369 |
| Age (reference : *21-30*) | | | | |
| *31-40* |  |  | 1.62 [1.10-3.37] | *0.014* |
| *41≤* |  |  | 1.27 [0.96–1.69] | 0.100 |
| Experience (reference : *1-5*) | | | | |
| *6-10* |  |  | 0.56 [0.40-0.79] | *0.001* |
| *>10* |  |  | 0.82 [0.63–1.07] | 0.143 |
| Hours worked per week (reference : *≤44*) |  |  | 0.70 [0.58–0.85] | *<0.001* |
| Education in nursing (reference : *Bachelor degree*) |  |  | 0.51 [0.40–0.64] | *<0.001* |
| Current work unit (reference : *Critical care units*) | | | | |
| *Emergency department* |  |  | 1.39 [1.11–1.72] | *0.004* |
| *General wards* |  |  | 0.51 [0.40–0.64] | *0.001* |
| Number of beds (reference : *200>*) | | | | |
| *200-499* |  |  | 0.98 [0.76–1.27] | 0.880 |
| *500≤* |  |  | 0.87 [0.68–1.12] | 0.269 |
| **Patient fall** | | | | |
| Supervisor Expectation & Actions Promoting Safety | 0.92 [0.80-1.07] | *0.294* | 0.91 [0.78-1.06] | 0.238 |
| Organizational Learning-Continuous Improvement | 0.72 [0.62-0.83] | *<0.001* | 0.75 [0.64-0.87] | *<0.001* |
| Teamwork Within Units | 1.18 [1.03-1.36] | *0.021* | 1.16 [1.01-1.35] | 0.041 |
| Communication Openness | 0.99 [0.85-1.15] | *0.861* | 0.98 [0.83-1.15] | 0.773 |
| Feedback and Communication About Error | 1.10 [0.95-1.27] | *0.205* | 1.14 [0.98-1.33] | 0.092 |
| Non-punitive Response to Error | 1.11 [0.99-1.24] | *0.080* | 1.14 [1.01-1.28] | 0.036 |
| Staffing | 0.76 [0.65-0.88] | *<0.001* | 0.74 [0.64-0.87] | *<0.001* |
| Hospital Management Support for Patient Safety | 0.79 [0.68-1.93] | *0.003* | 0.75 [0.64-0.88] | *0.001* |
| Teamwork Across Hospital Units | 0.86 [0.73-1.06] | *0.162* | 0.84 [0.69-1.02] | 0.077 |
| Hospital Handoffs and Transitions | 1.11 [0.98-1.26] | *0.093* | 1.10 [0.96-1.25] | 0.169 |
| Overall Perceptions of Safety | 1.08 [0.91-1.28] | *0.399* | 1.06 [0.88-1.26] | 0.557 |
| Frequency of Event Reporting | 0.85 [0.75-0.95] | *0.006* | 0.88 [0.78-0.99] | 0.044 |
| Gender (reference : *Male*) |  |  | 1.27 [1.02-1.59] | *0.031* |
| Marital status (reference : *Single*) |  |  | 1.07 [0.88-1.30] | 0.479 |
| Age (reference : *21-30*) | | | | |
| *31-40* |  |  | 0.94 [0.65-1.38] | 0.759 |
| *41≤* |  |  | 0.98 [0.74-1.30] | 0.890 |
| Experience (reference : *1-5*) | | | | |
| *6-10* |  |  | 0.93 [0.67-1.30] | 0.687 |
| *>10* |  |  | 0.98 [0.76-1.29] | 0.934 |
| Hours worked per week (reference : *≤44*) |  |  | 0.79 [0.66-0.96] | 0.018 |
| Education in nursing (reference : *Bachelor degree*) |  |  | 0.42 [0.34-0.53] | *<0.001* |
| Current work unit (reference : *Critical care units*) | | | | |
| *Emergency department* |  |  | 0.44 [0.35-0.54] | *<0.001* |
| *General wards* |  |  | 1.06 [0.84-1.34] | 0.604 |
| Number of beds (reference : *200>*) | | | | |
| *200-499* |  |  | 0.66 [0.51-0.86] | 0.002 |
| *500≤* |  |  | 0.52 [0.41-0.67] | *<0.001* |
| **Adverse Drug Events** | | | | |
| Supervisor Expectation & Actions Promoting Safety | 0.77 [0.66-0.90] | *0.001* | 0.79 [0.68-0.93] | *0.005* |
| Organizational Learning-Continuous Improvement | 0.83 [0.81-0.97] | *0.018* | 0.86 [0.73-1.00] | 0.054 |
| Teamwork Within Units | 1.28 [1.11-1.49] | *0.001* | 1.29 [1.11-1.50] | 0.001 |
| Communication Openness | 0.80 [0.68-0.94] | *0.007* | 0.78 [0.68-0.94] | 0.007 |
| Feedback and Communication About Error | 1.12 [0.97-1.31] | *0.128* | 1.05 [0.90-1.23] | 0.545 |
| Non-punitive Response to Error | 1.49 [1.32-1.68] | *<0.001* | 1.46 [1.29-1.65] | *<0.001* |
| Staffing | 0.79 [0.68-0.93] | *0.003* | 0.77 [0.65-0.89] | 0.001 |
| Hospital Management Support for Patient Safety | 1.02 [0.87-1.20] | *0.807* | 1.02 [0.86-1.12] | 0.862 |
| Teamwork Across Hospital Units | 1.10 [0.90-1.33] | *0.358* | 1.12 [0.92-1.37] | 0.263 |
| Hospital Handoffs and Transitions | 0.76 [0.66-0.86] | *<0.001* | 0.79 [0.69-0.90] | *<0.001* |
| Overall Perceptions of Safety | 1.02 [0.85-1.22] | *0.853* | 0.98 [0.82-1.18] | 0.841 |
| Frequency of Event Reporting | 0.76 [0.67-0.86] | *<0.001* | 0.78 [0.68-0.88] | *<0.001* |
| Gender (reference : *Male*) |  |  | 1.52 [1.21-1.91] | *<0.001* |
| Marital status (reference : *Single*) |  |  | 0.93 [0.77-1.13] | 0.477 |
| Age (reference : *21-30*) | | | | |
| *31-40* |  |  | 0.89 [0.61-1.32] | 0.580 |
| *41≤* |  |  | 0.87 [0.65-1.17] | 0.361 |
| Experience (reference : *1-5*) | | | | |
| *6-10* |  |  | 0.76 [0.54-1.06] | 0.109 |
| *>10* |  |  | 0.92 [0.70-1.21] | 0.557 |
| Hours worked per week (reference : *≤44*) |  |  | 0.95 [0.78-1.15] | 0.613 |
| Education in nursing (reference : *Bachelor degree*) |  |  | 0.68 [0.54-0.86] | *0.001* |
| Current work unit (reference : *Critical care units*) | | | | |
| *Emergency department* |  |  | 1.00 [0.81-1.25] | *0.963* |
| *General wards* |  |  | 0.84 [0.67-1.06] | 0.149 |
| Number of beds (reference : *200>*) | | | | |
| *200-499* |  |  | 1.55 [1.19-2.01] | 0.001 |
| *500≤* |  |  | 1.01 [0.79-1.30] | *0.914* |
| **Surgical wound infection** | | | | |
| Supervisor Expectation & Actions Promoting Safety | 0.83 [0.71-0.96] | 0.015 | 0.86 [0.73-1.00] | 0.054 |
| Organizational Learning-Continuous Improvement | 0.94 [0.81-1.09] | 0.388 | 0.98 [0.84-1.14] | 0.750 |
| Teamwork Within Units | 1.14 [0.99-1.31] | 0.078 | 1.13 [0.98-1.31] | 0.090 |
| Communication Openness | 0.80 [0.69-0.94] | 0.006 | 0.79 [0.68-0.93] | 0.004 |
| Feedback and Communication About Error | 1.05 [0.91-1.22] | 0.510 | 0.99 [0.86-1.16] | 0.956 |
| Non-punitive Response to Error | 1.36 [1.21-1.53] | <0.001 | 1.35 [1.20-1.52] | <0.001 |
| Staffing | 0.81 [0.70-0.94] | 0.004 | 0.78 [0.67-0.91] | 0.002 |
| Hospital Management Support for Patient Safety | 0.81 [0.69-0.95] | 0.009 | 0.80 [0.68-0.94] | 0.007 |
| Teamwork Across Hospital Units | 0.96 [0.80-1.16] | 0.680 | 0.95 [0.78-1.16] | 0.616 |
| Hospital Handoffs and Transitions | 0.84 [0.74-0.95] | 0.005 | 0.86 [0.76-0.98] | 0.025 |
| Overall Perceptions of Safety | 1.18 [0.99-1.40] | 0.060 | 1.16 [0.98-1.39] | 0.092 |
| Frequency of Event Reporting | 0.78 [0.69-0.88] | <0.001 | 0.77 [0.68-0.88] | <0.001 |
| Gender (reference : *Male*) |  |  | 1.29 [1.04-1.62] | *0.020* |
| Marital status (reference : *Single*) |  |  | 1.08 [0.89-1.31] | 0.425 |
| Age (reference : *21-30*) | | | | |
| *31-40* |  |  | 1.13 [0.77-1.64] | 0.540 |
| *41≤* |  |  | 0.89 [0.67-1.18] | 0.431 |
| Experience (reference : *1-5*) | | | | |
| *6-10* |  |  | 0.73 [0.53-1.02] | 0.063 |
| *>10* |  |  | 1.03 [0.70-0.79] | 0.806 |
| Hours worked per week (reference : *≤44*) |  |  | 0.95 [0.78-1.14] | 0.573 |
| Education in nursing (reference : *Bachelor degree*) |  |  | 0.66 [0.53-0.83] | <0.001 |
| Current work unit (reference : *Critical care units*) | | | | |
| *Emergency department* |  |  | 0.66 [0.53-0.81] | <0.001 |
| *General wards* |  |  | 0.73 [0.67-1.06] | 0.007 |
| Number of beds (reference : *200>*) | | | | |
| *200-499* |  |  | 1.27 [0.98-1.64] | 0.070 |
| *500≤* |  |  | 0.69 [0.54-0.88] | *0.003* |
| **Infusion or transfusion reaction** | | | | |
| Supervisor Expectation & Actions Promoting Safety | 0.82 [0.71-0.96] | 0.011 | 0.84 [0.72-0.97] | 0.022 |
| Organizational Learning-Continuous Improvement | 0.88 [0.76-1.02] | 0.097 | 0.92 [0.79-1.07] | 0.299 |
| Teamwork Within Units | 1.12 [0.97-1.29] | 0.121 | 1.10 [0.96-1.27] | 0.180 |
| Communication Openness | 0.91 [0.78-1.06] | 0.206 | 0.91 [0.78-1.07] | 0.242 |
| Feedback and Communication About Error | 0.96 [0.83-1.11] | 0.572 | 0.94 [0.81-1.09] | 0.413 |
| Non-punitive Response to Error | 1.25 [1.12-1.40] | <0.001 | 1.23 [1.09-1.38] | 0.001 |
| Staffing | 1.03 [0.89-1.19] | 0.740 | 1.02 [0.88-1.18] | 0.813 |
| Hospital Management Support for Patient Safety | 0.96 [0.82-1.12] | 0.601 | 0.94 [0.81-1.10] | 0.454 |
| Teamwork Across Hospital Units | 0.87 [0.72-1.05] | 0.153 | 0.88 [0.73-1.07] | 0.192 |
| Hospital Handoffs and Transitions | 0.80 [0.71-0.90] | <0.001 | 0.82 [0.73-0.94] | 0.003 |
| Overall Perceptions of Safety | 0.96 [0.81-1.15] | 0.684 | 0.93 [0.78-1.11] | 0.442 |
| Frequency of Event Reporting | 0.74 [0.65-0.83] | <0.001 | 0.75 [0.66-0.85] | <0.001 |
| Gender (reference : *Male*) |  |  | 1.17 [0.94-1.45] | *0.159* |
| Marital status (reference : *Single*) |  |  | 1.09 [0.91-1.33] | 0.364 |
| Age (reference : *21-30*) | | | | |
| *31-40* |  |  | 1.22 [0.84-1.77] | 0.291 |
| *41≤* |  |  | 1.13 [0.86-1.50] | 0.383 |
| Experience (reference : *1-5*) | | | | |
| *6-10* |  |  | 0.71 [0.52-1.99] | 0.041 |
| *>10* |  |  | 0.92 [0.71-1.19] | 0.542 |
| Hours worked per week (reference : *≤44*) |  |  | 0.85 [0.70-1.02] | 0.081 |
| Education in nursing (reference : *Bachelor degree*) |  |  | 0.54 [0.43-0.67] | *<0.001* |
| Current work unit (reference : *Critical care units*) | | | | |
| *Emergency department* |  |  | 1.02 [0.83-1.26] | *0.858* |
| *General wards* |  |  | 1.01 [0.81-1.27] | 0.913 |
| Number of beds (reference : *200>*) | | | | |
| *200-499* |  |  | 1.17 [0.91-1.51] | 0.092 |
| *500≤* |  |  | 0.81 [0.64-1.04] | <0.001 |
| **Patients or their family complaints** | | | | |
| Supervisor Expectation & Actions Promoting Safety | 0.69 [0.59-0.81] | <0.001 | 0.73 [0.62-0.85] | <0.001 |
| Organizational Learning-Continuous Improvement | 0.93 [0.79-1.08] | 0.343 | 0.97 [0.83-1.14] | 0.705 |
| Teamwork Within Units | 1.08 [0.93-1.26] | 0.303 | 1.07 [0.92-1.25] | 0.393 |
| Communication Openness | 0.84 [0.71-0.98] | 0.032 | 0.84 [0.71-0.99] | 0.034 |
| Feedback and Communication About Error | 1.12 [0.97-1.31] | 0.134 | 1.06 [0.90-1.24] | 0.507 |
| Non-punitive Response to Error | 1.39 [1.23-1.57] | <0.001 | 1.35 [1.12-1.53] | <0.001 |
| Staffing | 0.87 [0.75-1.02] | 0.086 | 0.83 [0.71-0.98] | 0.026 |
| Hospital Management Support for Patient Safety | 0.95 [0.81-1.12] | 0.555 | 0.92 [0.78-1.09] | 0.324 |
| Teamwork Across Hospital Units | 1.08 [0.88-1.31] | 0.475 | 1.11 [0.91-1.36] | 0.313 |
| Hospital Handoffs and Transitions | 0.68 [0.59-0.77] | <0.001 | 0.69 [0.60-0.79] | <0.001 |
| Overall Perceptions of Safety | 1.25 [1.04-1.50] | 0.016 | 1.12 [1.00-1.44] | 0.055 |
| Frequency of Event Reporting | 0.82 [0.72-0.93] | 0.002 | 0.85 [0.74-0.97] | 0.015 |
| Gender (reference : *Male*) |  |  | 1.28 [1.01-1.61] | *0.040* |
| Marital status (reference : *Single*) |  |  | 1.01 [0.83-1.24] | 0.895 |
| Age (reference : *21-30*) | | | | |
| *31-40* |  |  | 1.28 [0.86-1.89] | 0.228 |
| *41≤* |  |  | 0.89 [0.66-1.21] | 0.471 |
| Experience (reference : *1-5*) | | | | |
| *6-10* |  |  | 0.54 [0.38-0.76] | <0.001 |
| *>10* |  |  | 0.78 [0.59-1.04] | 0.086 |
| Hours worked per week (reference : *≤44*) |  |  | 0.57 [0.61-0.92] | 0.005 |
| Education in nursing (reference : *Bachelor degree*) |  |  | 0.46 [0.36-0.59] | *<0.001* |
| Current work unit (reference : *Critical care units*) | | | | |
| *Emergency department* |  |  | 0.72 [0.57-0.89] | *0.004* |
| *General wards* |  |  | 0.86 [0.68-1.09] | 0.207 |
| Number of beds (reference : *200>*) | | | | |
| *200-499* |  |  | 1.42 [1.09-1.85] | 0.010 |
| *500≤* |  |  | 0.99 [0.79-1.28] | *0.956* |
